# Supplementary material for: PHYTOCHROME-INTERACTING FACTOR 4/HEMERA-mediated thermosensory growth requires the Mediator subunit MED14
Source: Plant Physiol. 2022 Sep 5;190(4):2706–21. doi: 10.1093/plphys/kiac412 (PMC9706435; doi:10.1093/plphys/kiac412)
Supplement: kiac412_Supplementary_Data [file kiac412_supplementary_data.zip › kiac412_Supplementary_Data/Supplemental Data.pdf]

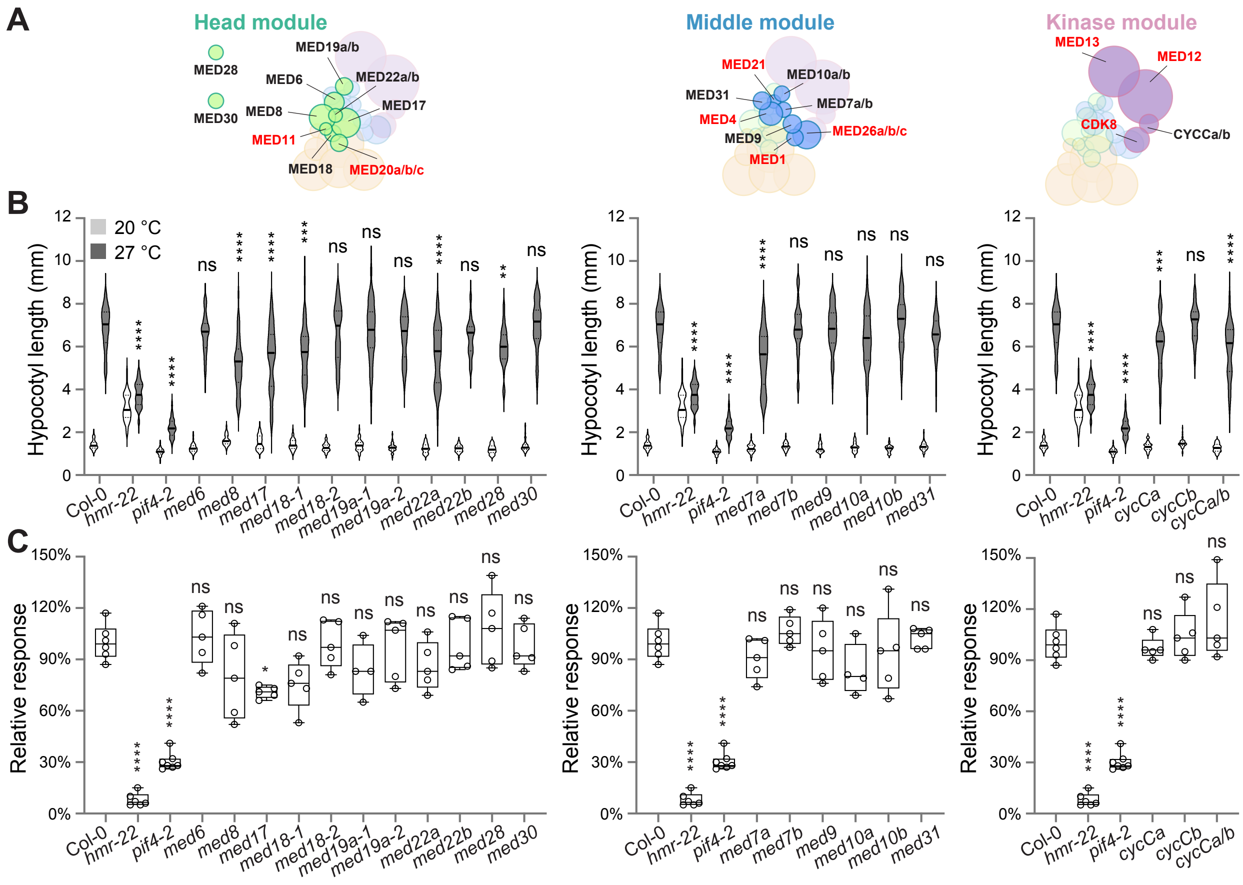

Supplemental Figure S1. Thermomorphogenetic hypocotyl responses in mediator mutants.

(A) Schematic illustration of the Arabidopsis Mediator subunits. The size of each circle reflects the relative protein size (predicted molecular weight) of each mediator subunit. The relative position of each mediator component is based on the cryo-EM and crystal structures of yeast and human Mediator (Tsai et al., 2014; Robinson et al., 2015) and interaction data of Arabidopsis mediator subunits (Maji et al., 2019). Protein subunits in each module are colored in different colors: head module, green; middle module, blue; tail module, orange; cyclin kinase module, purple. Null mutants of the mediator subunits labeled with red were not available or lethal.

(B) Hypocotyl length measurements of mutant seedlings of mediator head, middle, and kinase subunits. Seedlings were grown for 4 days in continuous red (R) light (50 µmol m^{-2} s^{-1}). The white and grey violin plots represent hypocotyl length measurements at 20 °C and 27 °C, respectively. The elements of violin plots are as follows: solid line, median; lower dotted line, first quartile; upper dotted line, third quartile. The results of the one-way ANOVA analysis comparing the absolute hypocotyl length between Col-0 and each mutant grown at 27 °C are shown (n > 30). ****, $p < 0.0001$; ***, $p < 0.001$; **, $p < 0.01$; *, $p < 0.05$; ns, not significant ($p \geq 0.05$).

(C) Comparison of the relative thermal response among the seedlings in (B). The relative response is defined as the relative hypocotyl response to 27 °C of a mutant compared with that of Col-0 (which is set at 100%). The elements of box plots are as follows: center line, median; box limits, first and third quartiles; whiskers, minimum and maximum values; points, all data points. The results of the one-way ANOVA analysis comparing the relative response between Col-0 and each mutant are shown (n ≥ 5). ****, $p < 0.0001$; ***, $p < 0.001$; **, $p < 0.01$; *, $p < 0.05$; ns, not significant ($p \geq 0.05$).

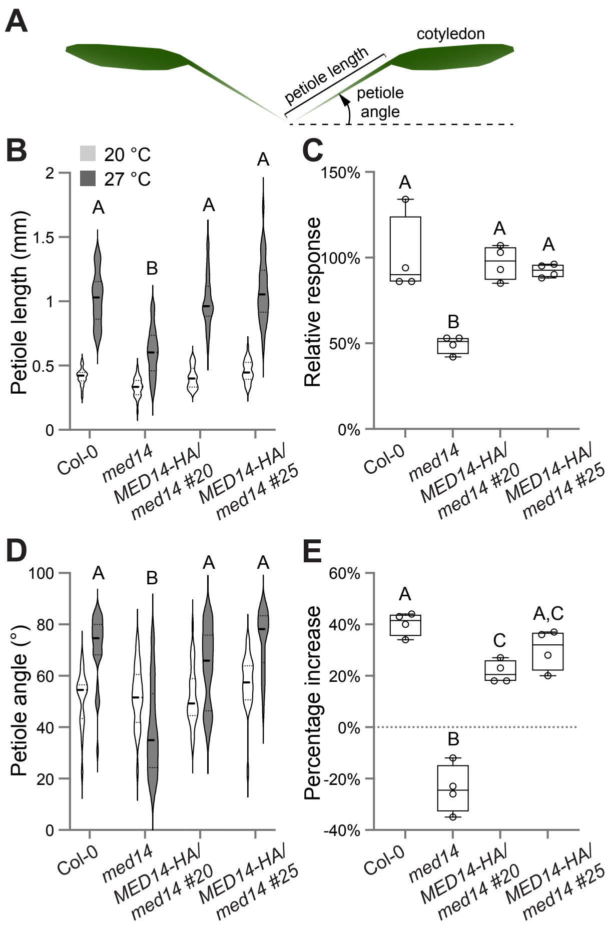

Supplemental Figure S2. MED14 regulates warm temperature-induced petiole elongation and leaf hyponastic response.

(A) An illustration showing how petiole length and angle are measured.

(B) Petiole length measurements of 4-d-old Col-0, *med14*, and *35S::MED14-HA/med14* (#20 and #25) seedlings grown in continuous red (R) light (50 µmol m^{-2} s^{-1}) at either 20 °C or 27 °C. The elements of violin plots are as follows: solid line, median; lower dotted line, first quartile; upper dotted line, third quartile. Different letters denote significant statistical differences between the absolute petiole length of each line grown at 27 °C (one-way ANOVA, n ≥ 30, $p < 0.0001$).

(C) Comparison of the relative thermal response among the seedlings in (B). The elements of box plots are as follows: center line, median; box limits, first and third quartiles; whiskers, minimum and maximum values; points, all data points. Different letters denote significant statistical differences between the relative response of each line (one-way ANOVA, n = 4, $p < 0.01$).

(D) Petiole angle measurements of 4-d-old Col-0, *med14*, and *35S::MED14-HA/med14* (#20 and #25) seedlings grown in continuous R light (50 µmol m^{-2} s^{-1}) at either 20 °C or 27 °C. The elements of violin plots are as follows: solid line, median; lower dotted line, first quartile; upper dotted line, third quartile. Different letters denote significant statistical differences between the petiole angle of each line grown at 27 °C (one-way ANOVA, n ≥ 18, $p < 0.0001$).

(E) Comparison of the percentage increase of petiole angle between seedlings grown at 20 °C and 27 °C in (D). The elements of box plots are as follows: center line, median; box limits, first and third quartiles; whiskers, minimum and maximum values; points, all data points. Different letters denote significant statistical differences between the relative response of each line (one-way ANOVA, n = 4, $p < 0.05$).

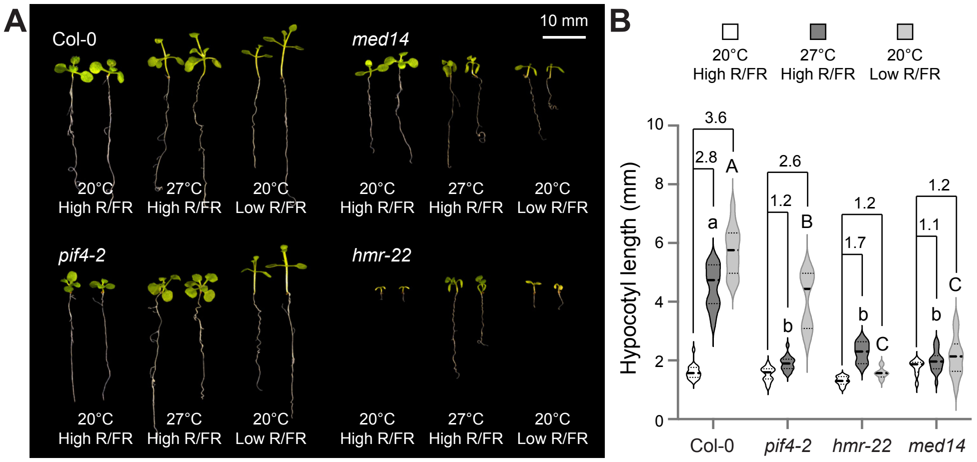

Supplemental Figure S3. MED14 is required for both warm temperature- and shade-induced hypocotyl elongation.

(A) Representative images of 12-d-old Col-0, *pif4-2*, *hmr-22*, and *med14* seedlings grown in continuous white light (30 μmol m^{-2} s^{-1}) at 20 °C with a high red to far-red (R/FR) ratio (10.2), 27 °C with a high R/FR ratio (10.2), and 20 °C with a low R/FR ratio (0.4). Images of seedlings grown in different conditions were digitally extracted and assembled for easy comparison. All the images were acquired using a Leica MZ10 F modular stereo microscope with the same settings (e.g., light, focus, and magnification). Scale bar, 10 mm.

(B) Hypocotyl length measurements of seedlings in (A). The elements of violin plots are as follows: solid line, median; lower dotted line, first quartile; upper dotted line, third quartile. Different lowercase and uppercase letters denote significant statistical differences between the absolute hypocotyl length of each line grown at 27 °C with a high R/FR ratio and 20 °C with a low R/FR ratio, respectively (one-way ANOVA, n ≥ 3, $p < 0.0001$). The numbers show the fold differences between hypocotyl length at 27 °C with a high R/FR ratio (dark grey) or 20 °C with a low R/FR ratio (light grey) and 20 °C with a high R/FR ratio (white).

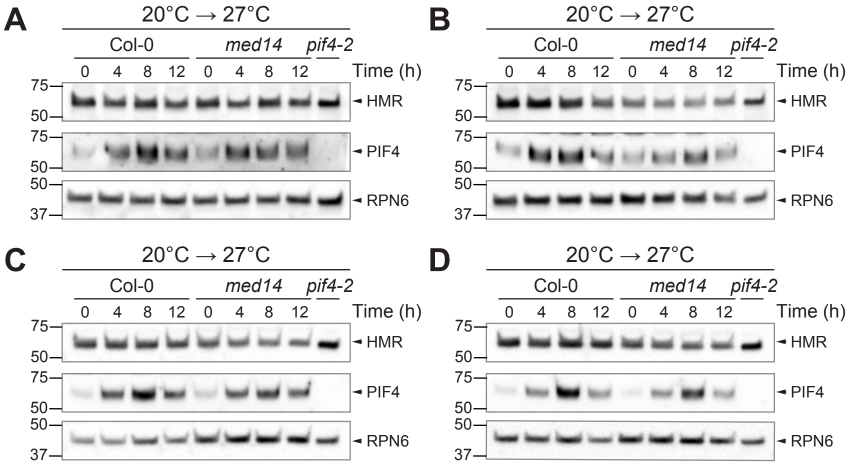

E **HMR Protein Level**

	RepA	RepB	RepC	RepD	AVG	SD
C,0h	1.00	1.00	1.00	1.00	1.00	0.00
C,4h	0.75	0.86	0.97	0.92	0.88	0.10
C,8h	0.97	0.86	0.65	1.03	0.88	0.17
C,12h	0.70	0.61	0.74	1.34	0.85	0.33
m,0h	0.98	0.44	0.45	0.73	0.65	0.26
m,4h	0.68	0.43	0.32	0.61	0.51	0.16
m,8h	0.96	0.41	0.28	0.54	0.55	0.29
m,12h	0.63	0.61	0.30	0.68	0.55	0.17

F **PIF4 Protein Level**

	RepA	RepB	RepC	RepD	AVG	SD
C,0h	1.00	1.00	1.00	1.00	1.00	0.00
C,4h	3.64	4.65	4.84	3.84	4.24	0.59
C,8h	6.69	6.10	5.18	7.20	6.29	0.87
C,12h	3.91	3.44	3.15	4.96	3.87	0.80
m,0h	2.46	0.78	0.59	0.63	1.11	0.90
m,4h	4.97	2.06	2.00	2.73	2.94	1.39
m,8h	4.96	2.64	2.51	5.34	3.86	1.49
m,12h	3.09	2.52	2.14	3.06	2.70	0.46

Supplemental Figure S4. Quantification of HMR and PIF4 protein levels during thermal treatments.

(A-D) Immunoblots of HMR and PIF4 levels in Col-0 and *med14* during the 20 ^{o}C-to-27 ^{o}C transitions. Seedlings were grown in continuous R light (50 μmol m^{-2} s^{-1}) at 20 ^{o}C for 96 h and then transferred to 27 ^{o}C in the same light condition. Samples were collected and analyzed at the indicated time points. RPN6 was used as a loading control. (C) was used as a representative result in Figure 2.

(E-F) The relative levels of HMR (E) and PIF4 (F), normalized to RPN6, are quantified based on the four immunoblots in (A-D). The value of Col-0 at 0 h (HMR/RPN6 or PIF4/RPN6) was set to 1.00 for each blot. Values were used to make the plots in Figure 2C. C, Col-0; m, *med14*; Rep, replicate; AVG, average; SD, standard deviation.

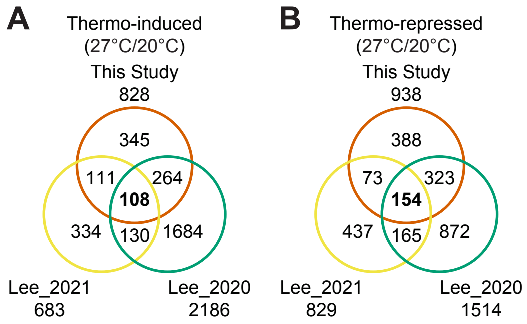

Supplemental Figure S5. Comparison of thermoresponsive transcriptomes in different studies. Venn diagrams are generated to compare the thermo-induced (A) and thermo-repressed (B) genes in the current study with those from another two studies (Lee 2020 and Lee 2021).

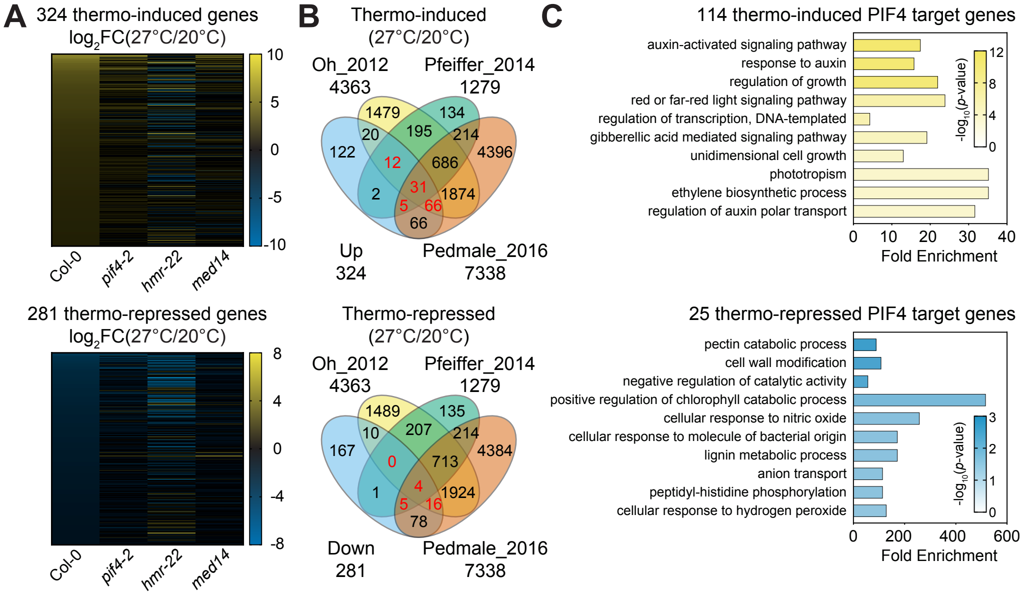

Supplemental Figure S6. Analysis of PIF4/HMR/MED14-dependent thermoresponsive genes.

(A) Heatmaps show the transcriptional patterns of 324 PIF4/HMR/MED14-dependent thermo-induced genes (upper panel) and 281 PIF4/HMR/MED14-dependent thermo-repressed genes (lower panel) in Col-0, *pif4-2*, *hmr-22*, and *med14* mutants.

(B) Venn diagrams show the overlapping genes between 324 PIF4/HMR/MED14-dependent thermo-induced genes (upper panel) or 281 PIF4/HMR/MED14-dependent thermo-repressed genes (lower panel) and PIF4-binding genes identified through chromatin immunoprecipitation (ChIP) assays in three previous studies (Oh et al., 2012; Pfeiffer et al., 2014; Pedmale et al., 2016). Red numbers indicate the thermo-regulated genes that were bound by PIF4 in at least two studies.

(C) Gene ontology analysis of the 114 PIF4/HMR/MED14-dependent thermo-induced PIF target genes (upper panel) and 25 PIF4/HMR/MED14-dependent thermo-repressed PIF4 target genes (lower panel). The bar represents the fold enrichment and the color indicates the -$\log_{10}$(p-value).

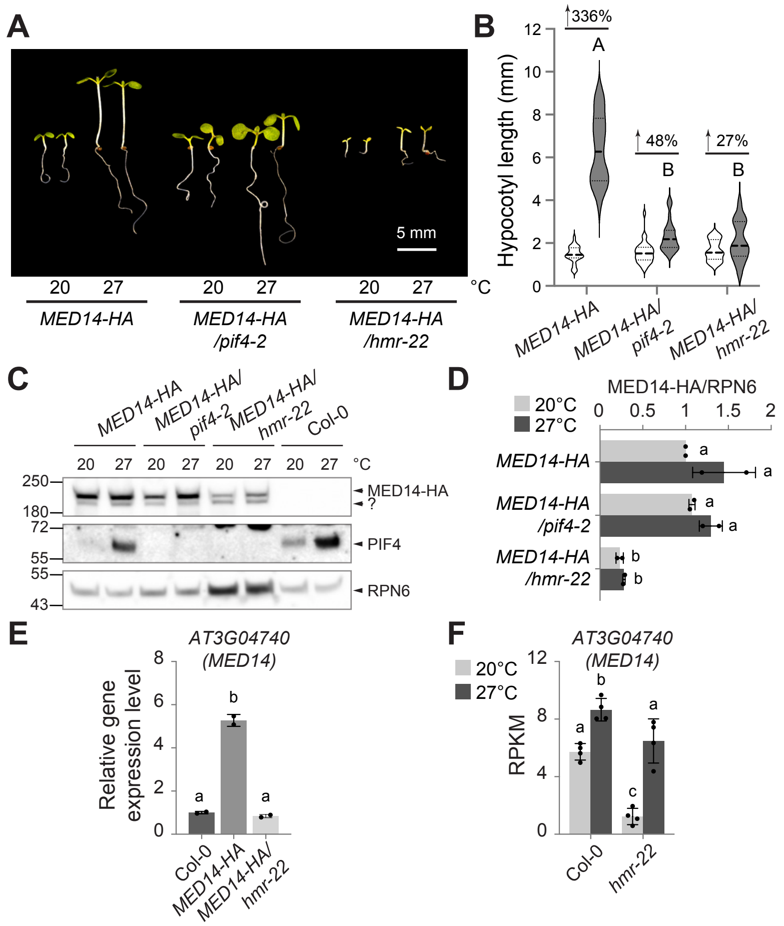

Supplemental Figure S7. Characterization of *35S::MED14-HA* lines crossed with *pif4-2* and *hmr-22*.

(A) Representative images of 4-d-old *35S::MED14-HA* (#20), *35S::MED14-HA/pif4-2*, and *35S::MED14-HA/hmr-22* seedlings in continuous red (R) light (50 μmol m^{-2} s^{-1}) at either 20 °C or 27 °C. Images of seedlings grown in different conditions were digitally extracted and assembled for easy comparison. All the images were acquired using a Leica MZ10 F modular stereo microscope with the same settings (e.g., light, focus, and magnification). Scale bar, 5 mm.

(B) Hypocotyl length measurements of seedlings in (A). The white and grey violin plots represent hypocotyl length measurements at 20 °C and 27 °C, respectively. The elements of violin plots are as follows: solid line, median; lower dotted line, first quartile; upper dotted line, third quartile. Different letters denote significant statistical differences between the absolute hypocotyl length of each line grown at 27 °C (one-way ANOVA, n > 15, $p < 0.0001$). The numbers and arrows indicate the percentage increase of hypocotyl length between 27 °C and 20 °C.

(C) Immunoblot analysis of MED14-HA and PIF4 levels in lines shown in (A). Seedlings were grown in continuous R light (50 μmol m^{-2} s^{-1}) at 20 °C for 96 h and then transferred to 27 °C or maintained at 20 °C in the same light condition. Samples were collected and analyzed after 6 h. Two bands were detected using anti-HA antibodies. The upper band was labeled as MED14-HA because it was close to the predicted protein size, while the identity of the lower band was unknown (?). PIF4 bands were detected using anti-PIF4 antibodies. RPN6 was used as a loading control.

(D) The relative levels of MED14-HA quantified from (C). The normalized level (MED14-HA/RPN6) in *35S::MED14-HA* at 20 °C was set to 1 and others were calculated as relative levels. The data were collected from two biological replicates (two technical replicates each) and different letters denote significant statistical differences (one-way ANOVA, n = 2, $p < 0.05$). Error bars represent the SD of two biological replicates.

(E) RT-qPCR analysis of *MED14* in Col-0, *35S::MED14-HA*, and *35S::MED14-HA/hmr-22* seedlings. Total RNA was extracted from seedlings grown in continuous R light (50 μmol m^{-2} s^{-1}) for 96 h at 20 °C and subsequently treated at 27 °C for 6 h. The relative expression of *MED14* was normalized to the expression level of *PP2A* after RT-qPCR. The transcript level in Col-0 was set to 1, and others were calculated by the $2^{-\Delta\Delta Ct}$ method. Error bars represent the SD of two biological replicates. Different letters denote significant statistical differences (one-way ANOVA, n = 2, $p < 0.001$).

(F) *MED14* transcript levels from RNA-seq data. RPKM (Reads Per Kilobase Million) values of *MED14* in 4-d-old Col-0 and *hmr-22* grown in continuous R light (50 μmol m^{-2} s^{-1}) at either 20 °C or 27 °C are compared. Different letters denote significant statistical differences (two-way ANOVA, n = 4, $p < 0.05$). Error bars represent the SD of four biological replicates.

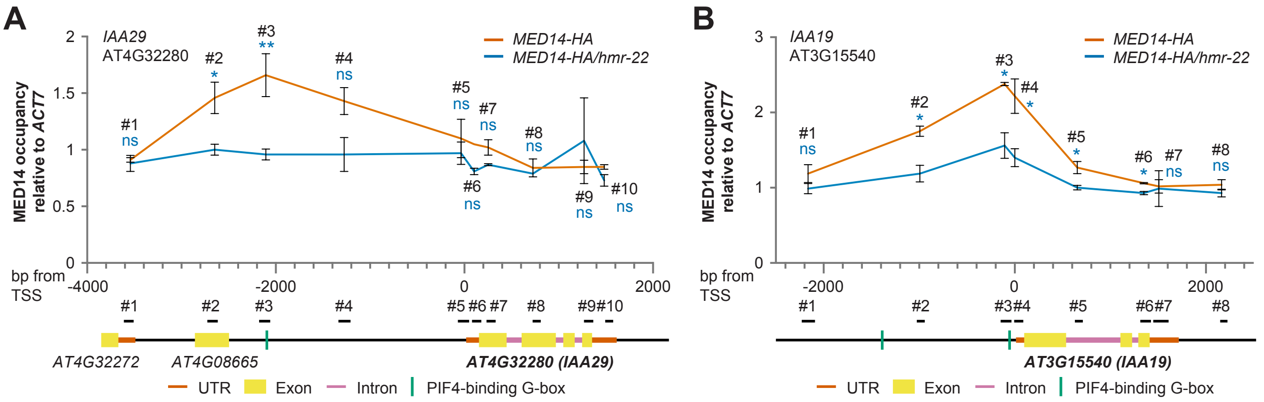

Supplemental Figure S8. The association of MED14 with thermo-induced PIF4 direct targets is reduced in the *hmr-22* mutant.

Chromatin immunoprecipitation (ChIP)-qPCR experiments compare MED14 occupancy across *IAA29* (A) or *IAA19* (B) in the presence and absence of functional HMR. *MED14-HA* and *MED14-HA/hmr-22* were grown in continuous R light (50 μmol m^{-2} s^{-1}) at 20 °C for 96 h and subsequently treated at 27 °C in the same light condition for 6 hours. Hemagglutinin (HA) antibodies were used to assay MED14-HA association with each gene. Values are mean ± SD from two independent samples, with data presented as the ratio of MED14-HA at *IAA*/input to MED14-HA at *ACT7* (+1912)/input. Student t-tests were used to compare the values between *MED14-HA* and other lines (n = 2). ****, $p < 0.0001$; ***, $p < 0.001$; **, $p < 0.01$; *, $p < 0.05$; ns, not significant ($p \geq 0.05$). The exact position of each PCR fragment is shown in the illustrations at the bottom and the primers are listed in Supplemental Table S4. The transcription start site (TSS) is the first nucleotide of the 5' untranslated region (UTR), as defined by the Araport11 genome annotation, released in June 2016. Please be noted that the reduced MED14-HA association with *IAA19* and *IAA29* promoters in the *hmr-22* background was probably caused by decreased PIF4 and MED14 levels.

Supplemental Table S1. List of T-DNA insertion lines and genotyping primers used in this study.

Accession	Gene name	Mutant name	Stock number	LP	RP[1]
AT1G11760	*MED2/29/32*	*med2-1*	SALK_023845C	TTTGCGGTATCAAATCGTTTC	CTCCACAAACTCCTCTGCTTG
AT1G11760	*MED2/29/32*	*med2-2*	SALK_028490C	TTTGCGGTATCAAATCGTTTC	CTCCACAAACTCCTCTGCTTG
AT3G09180	*MED3/27*	*med3*	SALK_012449	ACAATAACTTATGCCCCAGCC	GCAAATTAGAGAGATGCGTGG
AT3G23590	*MED5A/33A*	*med5a-1*	SALK_011621	TTGGTGCTGCCTATACCAGAC	ACCTAAGGAAACCATTGCCAC
AT3G23590	*MED5A/33A*	*med5a-2*	SALK_022477C	TTCAATAAGGTTTGCGACCAC	TTGCATTGGCAGATATTTTGC
AT2G48110	*MED5B/33B*	*med5b-1*	SALK_037472C	TGGGGTCAGTGACTGACTACC	TTCCCAGCTGTAAAGCAACTG
AT2G48110	*MED5B/33B*	*med5b-2*	SALK_015532	TTCTCTCTCTGTCGAGCTTGC	TCTTGGTTCATCTCGGTTCAC
AT3G21350	*MED6*	*med6*	SALK_055723	AGGAGGAGCTTACGAGAGGTG	TCTTCCAATGCCGTTTAGTTG
AT5G03220	*MED7A*	*med7a*	SALK_049958C	GAATGAATTCGGCAATTGATG	AATCGATGCAACAAACGATTC
AT5G03500	*MED7B*	*med7b*	SALK_001904	GGTTCAGGAGCAGAATCAGTG	TTGATTTAAGTCTTGGCGTCG
AT2G03070	*MED8*	*med8*	SALK_092406C	AATGTTAAAGAATGGGCCAGG	CTCAGCAAAAGTTTCAGACGG
AT1G55080	*MED9*	*med9*	SALK_115775	ATGTTGCTCTCGAAATCCATG	TGATTGTAACGACTGTGGCTG
AT5G41910	*MED10A*	*med10a*	SALK_115673C	CGAAAAGAGAAAGGGTTCGAG	GCAGGTGTGAAAGAAGAGACG
AT1G26665	*MED10B*	*med10b*	SALKSEQ_089446.2	ACCAATCTCTTCCTCCTCAGC	GTTCCCGCCTACTCAATAACC
AT3G04740	*MED14*	*med14*	SAIL_373_C07	GCTGCAACCAGACTTGATCTC	TTTGCTCAAAATGATTCCTGG
AT4G04920	*MED16*	*med16-2*	SALK_048091	TTGTACCAGAACTTGGCTTGG	CTCATCTCTTGCAGCTCCAAG
AT4G04920	*MED16*	*med16-3*	WISCDSLOX504A08	ACTCCGACCAAAAGACTCCAC	TTACCGCTGTTGCTTTTGATC
AT5G20170	*MED17*	*med17*	SALK_102813	TAAAGCATTGCGACCAGTACC	ACAAATTTTTCACGAGCATGC
AT2G22370	*MED18*	*med18-1*	SAIL_889_C08	AAGAAGCAAGTGGAGGAAGATG	CCCACATGGTACAGCTTCTTC
AT2G22370	*MED18*	*med18-2*	SALK_027178	TCCATCCGACATAAGAGATGC	TTCGGATTTGGTCTTGTTGAG
AT5G12230	*MED19A*	*med19a-1*	SALK_034955C	GAACGAGAAAAATTTGAGGCC	GCAAAAGAAATTCAGAGTGCG
AT5G12230	*MED19A*	*med19a-2*	SALK_020936	CAGGCAAGAGACTTGAGTTCG	AATAACGCAACATTTGCAAGG
AT1G16430	*MED22A*	*med22a*	SALK_063109C	AGAATCAGCTGCTTGAACCTG	TGCTGTTATTGGCCTTTTACG
AT1G07950	*MED22B*	*med22b*	SALK_001024C	TGGTTCAACGCGAATTAAATC	TTTGGTTTCGGTAGGTGAGTG
AT1G23230	*MED23*	*med23-1*	SALK_128011	CAGCAGCTCTAATCCGTTGAC	CGTTGTTGTGTTGTTGTTGTTG
AT1G23230	*MED23*	*med23-2*	SALK_060062	CTTCTCTTCAAGACTGCCGTG	TGGAGAACCAATACCATGAGC
AT1G25540	*MED25*	*med25-1*	SALK_060450	CCAAAAAGCACTGTCGATTTC	TCACAGCTCCAACATCATCAG
AT1G25540	*MED25*	*med25-2*	SALK_129555C	TGGAACTGGTCCAACAGAAAC	TGCATTGGCTTTCTTCCATAC
AT1G25540	*MED25*	*med25-3*	SALK_059316C	CGATCGAGTTGACCAAAGAAG	TTTGCATCAGGCAATATGTTG
AT3G52860	*MED28*	*med28*	SALK_037570	CCAGAGAACACTCTCAGGTGG	TCAAAGCTTCGGTTTCAACTG
AT5G63480	*MED30*	*med30*	SALK_117444	TGAAACCTTCATGAGATTGCC	AGCATCTGAAATGCAAAATGG
AT5G19910	*MED31*	*med31*	SALK_051025	CAACTTCAGGACTGCTATGGC	AACAAGTGCAACATGGAAACC
AT5G48630	*CYCCA*	*cycCa*	SAIL_102_B02	ATGACCCTTTGAACAATGCTG	GTAGCAAGTTTGTTGAAGGCG
AT5G48640	*CYCCB*	*cycCb*	SALK_053291C	AGAACCGTTGTCGTCGATATG	TGCCAATTTCTGGAATTCATC
AT5G48630/AT5G48640	*CYCCA/B*	*cycCa/cycCb*	SALK_039400C	TAATGTGTTGTGCCAGTTTCG	CTATTTTCCGTGTTTCAGGGG

[1]LP and RP are left and right genomic primers used for detecting the wild-type band; RP and a left border primer are used for detecting the T-DNA insertion. The left border primers for SALK lines, SAIL lines, and WiscDsLox collections are LBa1 (TGGTTCACGTAGTGGGCCATCG), LB3s (TAGCATCTGAATTTCATAACCA), and p745 (GTCCGCAATGTGTTATTAAGTTGTC), respectively.

Supplemental Table S2. Primers used for plasmid construction.

Accession	Gene name	Vector	Forward primer	Reverse primer
AT3G04740	*MED14*	pCHF1-$(PT)_4$P-3xHA	GAACACGGGGGACGAGCTATGGCGGAATTAGGGCAAC	TTGGTGTTGGAGTAGGAGCTATGGTAAATTCCTTTTGATGAG
AT3G04740	*MED14*	pCMX-PL2-NterHA	TCTCGAGAAGCTTGATATCGCTATGGCGGAATTAGGGCAAC	GCTACTAGCTAGCTGGCCAGACCTCTCTCTATATGGTAAATTCC
AT2G43010	*PIF4*	pET42b	GGGTCCATGGATATCGGGATGGAACACCAAGGTTGGAG	TGCTCGAGTGCGGCCGCAGCGTGGTCCAAACGAGAACCGTC
AT2G43010	*PIF4*	pDOE-01 (MCSI)	CAATTACATTTACAATTACATGGAACACCAAGGTTGGAG	CCCACCTCCGGATCCTCCAGCGTGGTCCAAACGAGAACCG
AT3G04740	*MED14*	pDOE-01/PIF4 (MCSIII)	CATTTACAATTACAATGGGATGGCGGAATTAGGGCAAC	CACCAGAACCTCCGGACGTTATGGTAAATTCCTTTTGATGAGTG

Supplemental Table S3. RT-qPCR primers for the genes examined in this study.

Accession	Gene name	Forward primer	Reverse primer
AT1G13320	*PP2A*	TATCGGATGACGATTCTTCGTGCAG	GCTTGGTCGACTATCGGAATGAGAG
AT2G43010	*PIF4*	AACCAGATCATCTCCGACCGGTTT	TCCCGCCGGTGAACTAAATCTCAA
AT2G34640	*HMR*	CCAGTAATTGTATTGTGCAGAGAC	CACTTACATCACCATCTCCATC
AT4G28720	*YUC8*	TGAAACAAAACAACCCACGA	TTGATTCGCTTTGGGTCTTC
AT3G15540	*IAA19*	ATCGGTGTGGCCTTGAAAG	AACATCCCCCAAGGTACATC
AT4G32280	*IAA29*	CACCATCATTGCCCGTATCA	CCACAGTAGCCGTTGTTGGA
AT5G07010	*ST2A*	TGCAACTTTCTTAGAGCTTCC	AGTTCTTGATCGACTTGTTTGAC
AT5G02760	*APD7*	CGTTATCCAGGTCACAAGATC	CGCACTAAGGATTGGCTTAG
AT3G59900	*ARGOS*	GAACAACCGAGTCATGGACG	AAACTCCGCCGTAACTCTTG
AT1G24580	*RING*	CCGAGACTGAATCTTCCATG	CACCTTTCTCTTCTTCTTCCTC
AT1G11080	*SCPL31*	AATTGGACTGGTTCAAACCC	CGCAAGTACTGGAACTCTTC
AT5G58310	*MES18*	CACCATCATTGCCCGTATCA	CCACAGTAGCCGTTGTTGGA
AT3G04740	*MED14*	AGTGACAAGACGGCATATCC	CGAAGCGAATTGCATCTTTC

Supplemental Table S4. ChIP-qPCR primers for the genes examined in this study.

Accession	Gene name	Number	Position[1]	Forward primer	Reverse primer
AT3G15540	*IAA19*	#1	+2163	CCAGATATTCACCTGATTCTCC	ATGTTCCCACGTCCATAAGC
AT3G15540	*IAA19*	#2	+992	TCTTTGGGAATTTGGTTTACC	CATATCATATGGATTGAATTTGTAC
AT3G15540	*IAA19*	#3	+104	CATCCTCAGTTGACCTGTCTC	AACCAATCCAATATCGACACG
AT3G15540	*IAA19*	#4	+1	TTCATTGGTTGTATCGTGTGG	AACTTTCTTCTCCTACACTTCTC
AT3G15540	*IAA19*	#5	+655	GAGTGTTGTAGTCTTGTAGACG	GGTGTTGGTTTGGTTGTAGAC
AT3G15540	*IAA19*	#6	+1350	CTAGAGTCATGCAAGAGGTTG	GCTCCTTGCTTCTTGTTCAAG
AT3G15540	*IAA19*	#7	+1509	TTGATCATCAAGATCCTTTAGAAC	CAACACTCAAGAAACAAGTAGTG
AT3G15540	*IAA19*	#8	+2164	TGCACATCTTGCTTTTGATAG	AACTAGGTTGACATACCGAAC
AT4G32280	*IAA29*	#1	-3541	CTCGTCTTCGTCTTCGTTCG	GTGTCCTCTACGGTCAGTATC
AT4G32280	*IAA29*	#2	-2648	AGACAGTAAGCCAAATCTGTC	TGGGAGACAAATAAGTGTTACG
AT4G32280	*IAA29*	#3	-2105	AGCCCATTACTTCAGTGGTC	CAACCAAGCAGAAGAGAGTAG
AT4G32280	*IAA29*	#4	-1274	GCAACTGATGAAGTCAATACTTG	AGTAATACTCCTAGCAAACTCTG
AT4G32280	*IAA29*	#5	-34	CCACATCTATATTTGTGTGGGTTAC	AGTAAAAGGAAATGGGGGCAG
AT4G32280	*IAA29*	#6	+103	CAGCAATCAACACCAACGAAC	CCATTTCTAAGGCAGCTTCGTC
AT4G32280	*IAA29*	#7	+253	ACTTTGACCTCAACAAGCATTG	ATTCCCTAACCCAAACGTCG
AT4G32280	*IAA29*	#8	+728	CACCATCATTGCCCGTATCA	CCACAGTAGCCGTTGTTGGA
AT4G32280	*IAA29*	#9	+1269	GGATCTTTGCGGAATCTGTTC	TCCGATTTGAACGCCTATCC
AT4G32280	*IAA29*	#10	+1483	CCATGGCTAACACATGGAC	ATCAGCTAGTCTGTCTTTGAC
AT5G09810	*ACT7*	#1	+1912	TGCTGACCGTATGAGCAAAG	TGATCCTCCGATCCAGACAC

[1]The position of each PCR fragment is defined as the distance (base pairs) between the central nucleotide of the fragment to the transcription start site (TSS) defined by the Araport11 genome annotation, released June 2016 (usually the first nucleotide of the 5'UTR). Positions upstream of the TSS are labeled "-" and downstream ones "+".
