# Supplementary figures and images for: PHYTOCHROME-INTERACTING FACTOR 4/HEMERA-mediated thermosensory growth requires the Mediator subunit MED14
Source: Plant Physiol. 2022 Sep 5;190(4):2706–21. doi: 10.1093/plphys/kiac412 (PMC9706435; doi:10.1093/plphys/kiac412)

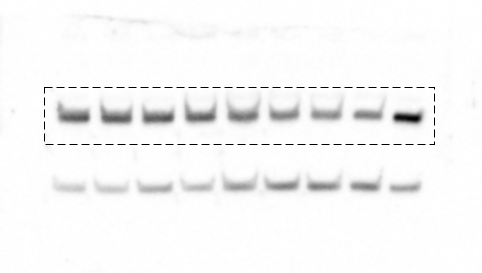

Supplement: kiac412_Supplementary_Data [file kiac412_supplementary_data.zip › kiac412_Supplementary_Data/SupFig4C_HMR_Source.tif]

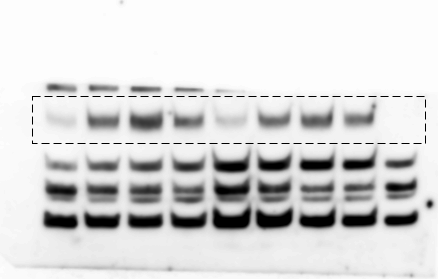

Supplement: kiac412_Supplementary_Data [file kiac412_supplementary_data.zip › kiac412_Supplementary_Data/SupFig4C_PIF4_Source.tif]

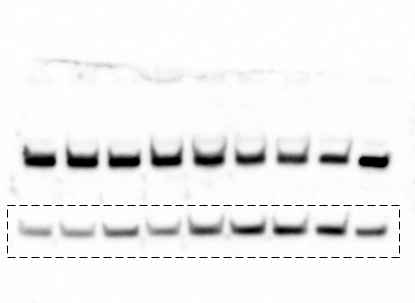

Supplement: kiac412_Supplementary_Data [file kiac412_supplementary_data.zip › kiac412_Supplementary_Data/SupFig4C_RPN6_Source.tif]

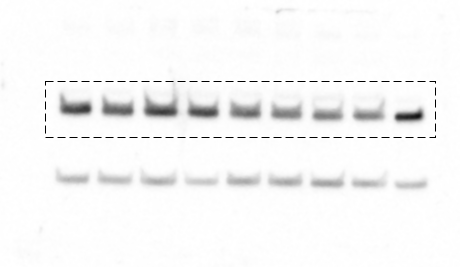

Supplement: kiac412_Supplementary_Data [file kiac412_supplementary_data.zip › kiac412_Supplementary_Data/SupFig4D_HMR_Source.tif]

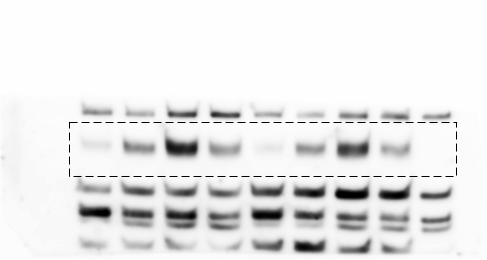

Supplement: kiac412_Supplementary_Data [file kiac412_supplementary_data.zip › kiac412_Supplementary_Data/SupFig4D_PIF4_Source.tif]

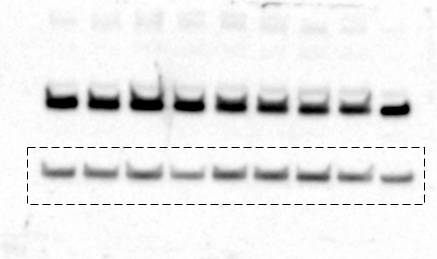

Supplement: kiac412_Supplementary_Data [file kiac412_supplementary_data.zip › kiac412_Supplementary_Data/SupFig4D_RPN6_Source.tif]
